# Quercetin Prevents *Escherichia coli* O157:H7 Adhesion to Epithelial Cells via Suppressing Focal Adhesions

**DOI:** 10.3389/fmicb.2018.03278

**Published:** 2019-01-16

**Authors:** Yansong Xue, Min Du, Mei-Jun Zhu

**Affiliations:** ^1^School of Food Science, Washington State University, Pullman, WA, United States; ^2^Department of Animal Sciences, Washington State University, Pullman, WA, United States

**Keywords:** *E. coli* O157:H7, quercetin, integrin β1, anti-adhesion, focal adhesion

## Abstract

The attachment of *Escherichia coli* O157:H7 to intestinal epithelial cells is indispensable for its pathogenesis. Besides translocated-intimin receptor (Tir), *E. coli* O157:H7 interacts with host cell surface receptors to promote intimate adhesion. This study showed that integrin β1 was increased in Caco-2 cells upon *E. coli* O157:H7 infection, while Caco-2 cells subjected to integrin β1 antibody blocking or CRISPR/Cas9 knockout had reduced bacterial attachment. Infection of *E. coli* O157:H7 inactivated focal adhesion kinase (FAK) and paxillin, increased focal adhesion (FA) and actin polymerization, and decreased cell migration in Caco-2 cells, which were rescued by integrin β1 antibody blocking or knockout. Pre-treatment with quercetin, known for its anti-oxidant and anti-inflammatory activity, reduced bacterial infection to Caco-2 cells, which might be partially via interfering integrin β1 and FAK association augmented by *E. coli* O157:H7. In addition, quercetin decreased FA formation induced by bacterial infection and recovered host cell motility. Taken together, data showed that *E. coli* O157:H7 interacts with integrin β1 to facilitate its adhesion to host cells. Quercetin inhibits bacterial infection possibly by blocking the interaction between *E. coli* O157:H7 and integrin β1. Collectively, these data indicate that quercetin provides an alternative antimicrobial to mitigate and control *E. coli* O157:H7 intestinal infection, and suggest potential broad benefits of quercetin and related polyphenols in fighting other enteric pathogen infections.

## Introduction

Formation intestinal attaching and effacing (A/E) lesions is of necessary for the pathogenesis of *Escherichia coli* O157:H7 (Kaper, 2005). After attachment to intestinal epithelial cells, *E. coli* O157:H7 induces actin rearrangement to form pedestals (Knutton et al., 1989). Through this tight association with the host cell surface, *E. coli* O157:H7 utilizes various strategies to manipulate host signaling, leading to enhanced bacterial colonization and persistence, and host tissue damage (Xue et al., 2017). The host extracellular matrix (ECM) is composed of multiple macromolecules, which mediate multiple biological functions including cell to cell adhesion, migration, proliferation, and death (Meredith et al., 1993). Integrin β1, the most abundant cell surface integrin, is a transmembrane glycoprotein receptor that interacts with ECM components such as fibronectin, laminin, and collagen. Through interactions with ECM components, integrin β1 induces multiple bidirectional signal exchanges (Schwartz et al., 1995; Burridge and Chrzanowska-Wodnicka, 1996). In addition, integrin β1 recruits intracellular proteins such as talin, paxillin, and α-actinin, leading to the formation of the focal adhesion (FA) complex.

To tightly associate with host cells, pathogens utilize integrin β1 as an adhesion factor. *Yersinia pseudotuberculosis* interacts with integrin β1 via adhesin YadA to promote tight binding to the host cells (Eitel et al., 2005). *Neisseria gonorrhoeae* attaches to ECM substrate with the assistance of host integrin β1 (Muenzner et al., 2005). In response to infection, the rapid turnover and exfoliation of epithelial cells are innate defense mechanisms against pathogens (Mulvey et al., 2000). However, many pathogenic bacteria can circumvent host exfoliation and colonize the epithelium efficiently. *Shigella flexneri* reduces adhesion complex turnover and suppresses the detachment of infected cells from the basement membrane to manipulate host exfoliation (Kim et al., 2009). Integrins transduce extracellular signals into the host cells through association with intracellular adaptor proteins and protein kinases such as focal adhesion kinase (FAK) (Dia and Gonzalez de Mejia, 2011) and integrin-linked kinase (ILK) (Gagne et al., 2010). FAK deficiency increases the recruitment of FAs and reduces cell motility (Ilic et al., 1995), indicating FAK is involved in FA formation during cell migration. Thus, pathogens may manipulate FAK and associated kinases, which stabilize the FAs and ultimately enable them to colonize the host cells.

Quercetin is a polyphenol widely found in vegetables and fruits. Our previous study demonstrated that quercetin had anti-inflammatory and anti-oxidative properties that prevented *E. coli* O157:H7-induced inflammasome activation (Xue et al., 2017). However, the antimicrobial mechanism of quercetin has not been elucidated. We hypothesized that *E. coli* O157:H7 attaches to host cells via interacting with host integrin β1 and stabilizing FAs formation; quercetin inhibits integrin β1 expression and FA formation thus preventing *E. coli* O157:H7 infection.

## Materials and Methods

### Cell Line, Media and Bacterial Strains

The human colonic epithelial cell line Caco-2 was obtained from the American Type Culture Collection (Manassas, VA, United States). Caco-2 cells were cultured in Dulbecco’s Modified Eagle’s medium (DMEM) (Sigma, St. Louis, MO, United States) supplemented with 10% fetal bovine serum (Sigma), 100 units/ml penicillin G, and 100 μg/ml of streptomycin (Sigma) at 37°C with 5% CO_2_. The *E. coli* O157:H7 EDL933 wild type (EDL933) strain was obtained from the STEC center at Michigan State University. The *E. coli* O157:H7 EDL933 intimin (Δ*eae*) and *tir* (Δ*tir*) mutant strains were kindly provided by Dr. Carolyn H. Bohach’s Lab at the University of Idaho. pEHEC *tir* plasmid was a generous gift from Dr. John M Leong at Tufts University (Campellone et al., 2002). EDL933Δ*tir* pEHEC *tir* strain was derived from *E. coli* O157:H7 EDL933Δ*tir* strain transformed with pEHEC *tir* plasmid. These strains were routinely grown in LB broth at 37°C overnight with aeration.

### Infection of *E. coli* O157:H7 to Colonic Epithelial Cells

Caco-2 cells were seeded in a 24-well plate at 5 × 10^5^ cells/ml for 12 h. Then the growth medium was replaced with fresh DMEM complete medium without antibiotics and supplemented with or without 200 μM quercetin (Sigma) for 12 h. Quercetin at this concentration did not impact the viability and growth of *E. coli* O157:H7 EDL933 (Supplementary Figure S1), nor did it decrease cell viability of Caco-2 cells (Xue et al., 2017). For integrin β1 blocking assay, cell monolayers were pretreated with integrin β1 antibody (rat IgG1, monoclonal, 1:200 dilution, DSHB) for 1 h prior infection, followed by 3 washes with PBS (pH 7.4). Then the cells were challenged with *E. coli* O157:H7 EDL933 at multiplicity of infection (MOI) of 10 for 4 h at 37°C with 5% CO_2_.

### Quantitative Reverse Transcription PCR (qRT-PCR) Analysis

Total RNA was extracted from Caco-2 cells with TRI Reagent (Sigma) and reverse transcribed using an iScript kit (Bio-Rad, Hercules, California). cDNAs were used as templates for qRT-PCR analysis of selected genes using a CFX96 Real-Time PCR Detection System (Bio-Rad). SYBR green master mix (Bio-Rad) was used for all qRT-PCR reactions. β-actin was used as the housekeeping gene. Primers for qRT-PCR are listed in Supplementary Table S1. Amplification efficiency was 0.90 to 0.99 (Xue et al., 2017).

### Immunoblotting

Immunoblotting analysis was conducted according to the procedures described (Xue et al., 2017). Antibodies against vinculin (mouse monoclonal IgG1), talin (mouse monoclonal IgG3), and α-actinin (mouse monoclonal IgG1) were purchased from Santa Cruz (Dallas, TX, United States). Anti-p-FAK (rabbit polyclonal), FAK (rabbit polyclonal), p-paxillin (rabbit polyclonal), paxillin (rabbit polyclonal), and integrin β1 (rabbit monoclonal IgG) antibodies were from Cell Signaling Technology (Beverly, MA, United States). Antibody against β-actin (mouse monoclonal IgG1) was purchased from DSHB (Iowa City, IA, United States). Binding of antibodies was detected using HRP-coupled anti-rabbit or anti-mouse immunoglobulin (Cell Signaling) and visualized using Pierce ECL Western blotting substrate (ThermoFisher Scientific, Waltham, MA, United States). Density of bands was quantified by ImageQuant TL software (GE Healthcare Life Sciences, PA) and then normalized with reference to the β-actin content.

### Adhesion of *E. coli* O157:H7 to Colonic Epithelial Cells

*Escherichia coli* O157:H7 attachment to Caco-2 cells was conducted as previously reported (Xue et al., 2017). Briefly, Caco-2 cells were seeded at 5 × 10^5^ cells/ml in a 24-well plate, cultured until 80∼90% confluence and treated with 0 or 200 μM quercetin for 12 h. The cell monolayers were next challenged with *E. coli* O157:H7 EDL933 strain (MOI = 10) and co-cultured at 37°C with 5% CO_2_ for 4 h, followed by 3 washes with ice cold PBS and lysed with 0.2% Triton X-100. Lysates were serially diluted and appropriate dilutions were plated on LB agar plates. The bacterial colonies were counted after 24 h incubation at 37°C.

### Immunofluorescent Staining

Cell culture, quercetin treatment, and infection procedure were conducted as described above. Post-infection, the cell monolayers were washed 3 times with ice cold PBS and fixed in fresh prepared 4% paraformaldehyde for 30 min at room temperature. The fixed cells were then permeabilized with 0.5% Triton X-100 for 10 min, washed with PBS, and blocked with 5% normal goat serum for 60 min at room temperature (RT). Then the cells were incubated with anti-integrin β1 antibody (rat monoclonal IgG1, DSHB), vinculin antibody (Santa Cruz) or phalloidin (Sigma) overnight at 4°C. The cells were rinsed with PBS and stained with Alexa Fluor 555 goat anti-rat IgG or Alexa Fluor 488 goat anti-mouse IgG (Cell Signaling) for 60 min at RT. These stained cells were washed 3 times with PBS and mounted with Fluoro-gel with DAPI (Electron Microscopy Sciences, Hatfield, PA). Fluorescence signal was visualized with EVOS FL fluorescence microscope (Life Technologies, Grand Island, NY).

### Co-immunoprecipitation

The post-infection cell monolayers were washed twice with ice-cold PBS and lysed in 200 μl IP buffer (50 mM Tris-HCl (pH 7.5), 150 mM NaCl, 1% (v/v) Triton X-100, 0.1% (w/v) Na-deoxycholate, 1 mM EDTA, proteinase inhibitor cocktail) for 15 min on ice. The resulting cell lysates were transferred into pre-cooled 1.5 ml tubes, passed through a 29-gauge needle twice, and centrifuged for 10 min at 14,000 g, 4°C. An aliquot of the supernatant was sampled for input protein content analysis. The remaining supernatants were pre-cleared with Protein G agarose beads (Thermo Scientific) with rotation for 30 min at 4°C. The pre-cleared supernatants were incubated with anti-integrin β1 antibody (rat monoclonal IgG1, 1:100, DSHB) overnight with rotation at 4°C. Then the Protein G magnetic agarose was added into the tubes and co-incubated overnight at 4°C with rotation. The next day, tubes were placed on the magnetic stand to collect beads. The beads were washed with IP buffer 5 times, then resuspended in 100 μl of loading buffer and heated to 100°C for 10 min to elute proteins. The supernatants collected were used for immunoblotting with anti-intimin-γ antibody (Gift from Dr. John M Leong) or anti-FAK antibody (Cell signaling), respectively.

### Integrin β1 CRISPR/Cas9 Knock Out (KO)

Caco-2 cells, at 70% confluence, were transfected with integrin β1 CRISPR/Cas9 KO plasmid (ITGB1 sgRNA/Cas9, GeneCopoeia, Rockville, MD, United States) or scramble control vector (Con sgRNA, pCRISPR-SG01, GeneCopoeia) using X-tremeGENE HP DNA transfection reagent (Sigma) per manufacturer’s instructions. Medium was changed 12 h post transfection, when 400 μg/ml G418 (Amresco, Solon, OH, United States) was added in the following 7 days to select cells with ITGB1 sgRNA.

### Cell Migration Activity

Cell culture, quercetin treatment, and infection procedure were conducted as described above. A scratch was introduced to the Caco-2 cell monolayer using a pipet tip. Then the cells were washed with PBS and infected with EDL933 strain or left uninfected for 4 h. Cells were washed with PBS and replaced with DMEM complete medium. Cells were migrated into the wound at 37°C for 24 h. The migration was assessed by counting the number of Caco-2 cells that crossed the wound border as published previously (Kung et al., 2008).

### Statistical Analyses

Statistical analyses were conducted as previously described (Xue et al., 2017). Data were analyzed as a complete randomized design using GLM (General Linear Model of Statistical Analysis System, SAS, 2000). All data were analyzed by two-tailed Student’s *t*-test. Means ± standard errors of mean (SEM) are reported. Statistical significance is considered as *P* ≤ 0.05.

## Results

### Integrin β1 Was Involved in *E. coli* O157:H7 Attachment

**FIGURE 1:**
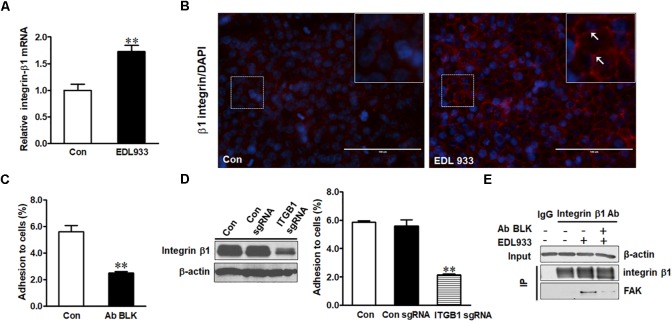
Integrin β1 mediated *E. coli* O157:H7 adhesion to Caco-2 cells. **(A)** mRNA expression of integrin β1. **(B)** Immunofluorescence staining for integrin β1. Blue, DAPI; Red, integrin β1. Images were taken at 200 × magnification. **(C)** Bacterial adhesion to Caco-2 cells with or without integrin β1 antibody blocking (Ab BLK). **(D)** Integrin β1 content and bacterial adhesion to Caco-2 cells transfected with scramble control (Con sgRNA) or integrin β1 knockout (ITGB1 sgRNA) plasmid. **(E)** Co-immunoprecipitation analysis of integrin β1 and FAK interaction. Input, the whole cell lysate detected with β-actin; IP, lysates post integrin β1 immunoprecipitation were detected with integrin β1 or FAK antibody. Caco-2 cells were co-cultured with EDL933 strain for 4 h before respective analyses. Means ± SEM; *n* = 4. ^∗∗^*P* ≤ 0.01.

Integrin β1 was expressed higher in infected cells than in control cells (Figure 1A). *E. coli* O157:H7 infection also increased surface level of integrin β1 (Figure 1B) as well as integrin α5 mRNA expression (Supplementary Figure S2). Neutralizing integrin β1 with anti-integrin β1 antibody reduced bacterial adhesion to Caco-2 cells (Figure 1C). To further explore the role of integrin β1 in bacterial adhesion, integrin β1 was knocked out with ITGB1 CRISPR/Cas9 sgRNA plasmid, which significantly attenuated EDL933 adherence to Caco-2 cells (Figure 1D). Integrin clustering is reported to be associated with FAK activation (Guan, 1997). Immunoprecipitation assay further showed FAK protein was associated with integrin β1 in Caco-2 cells infected with *E. coli* O157:H7, suggesting that infection induced FAK recruitment by integrin β1 (Figure 1E).

### Integrin β1 Was Implicated in Infection-Induced Dephosphorylation of FAK and Paxillin

**FIGURE 2:**
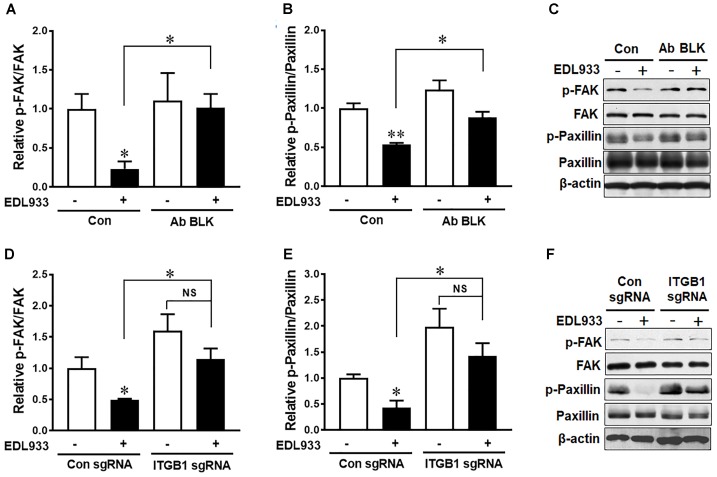
*Escherichia coli* O157:H7 inhibited FAK activation through interaction with integrin β1. **(A–C)** Immunoblotting analysis of FAK and paxillin in Caco-2 cells with or without integrin β1 antibody blocking (Ab BLK). **(D–F)** Phosphorylation of FAK and paxillin in Caco-2 cells transfected with scramble control (Con sgRNA) or integrin β1 knockout (ITGB1 sgRNA) plasmid followed with infection of EDL933 strain for 4 h. Means ± SEM; *n* = 4. ^∗∗^*P* ≤ 0.01; ^∗^*P* ≤ 0.05; NS, not significant at *P* ≤ 0.05.

FAK is a critical kinase that modulates FA activities (Zimerman et al., 2004). Phosphorylation of FAK and its downstream protein paxillin were markedly decreased in Caco-2 in response to *E. coli* O157:H7 infection (Figure 2). Anti-integrin β1 antibody blocking prevented dephosphorylation of FAK and paxillin induced by *E. coli* O157:H7 (Figures 2A–C). Similarly, with integrin β1 KO, *E. coli* O157:H7 infection could no longer cause FAK and paxillin dephosphorylation as compared with uninfected control (Figures 2D–F). These results indicated that integrin β1 had a mediatory role in *E. coli* O157:H7-induced FAK inhibition.

### Integrin β1 Increased FA and Actin Polymerization in Response to *E. coli* O157:H7 Infection

**FIGURE 3:**
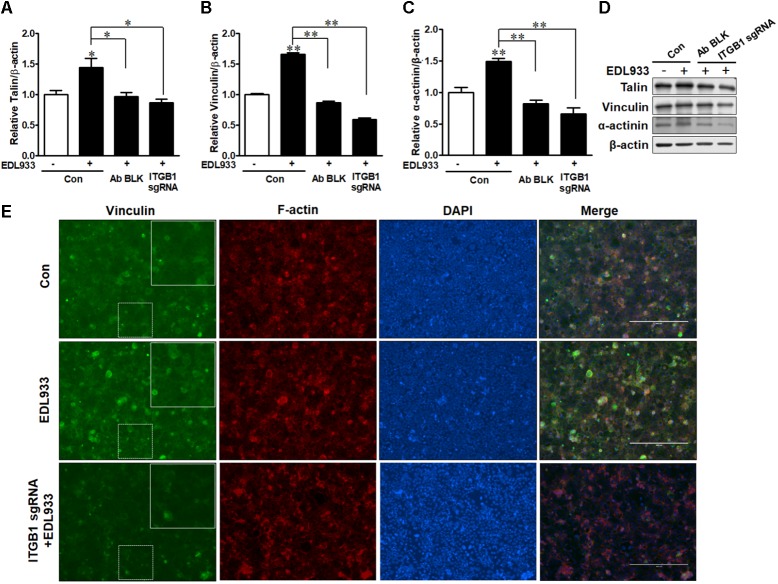
Focal adhesions were recruited upon *E. coli* O157:H7 infection in an integrin β1-dependent manner. **(A–D)** Immunoblotting analysis of talin, vinculin, and α-actinin. **(E)** Immunofluorescence staining for vinculin. Caco-2 cells were immuno-blocked with integrin β1 antibody (Ab BLK) or transfected with integrin β1 sgRNA (ITGB1 sgRNA) plasmid followed by infection with EDL933 strain for 4 h. Con. Caco-2 cells without infection; EDL933, Caco-2 cells infected with EDL933 strain; ITGB1 sgRNA+EDL933, integrin β1 knockout Caco-2 cells infected with EDL933 strain. Blue, DAPI; Red, F-actin; Green, vinculin. Images were taken at 100 × magnification. Means ± SEM; *n* = 4. ^∗∗^*P* ≤ 0.01; ^∗^*P* ≤ 0.05.

FA is responsible for cell adhesion and migration (Hu et al., 2014). Enhanced FA assembly reduces cell mobility (Wozniak et al., 2004). *E. coli* O157:H7 infection increased FA proteins including talin, vinculin, and α-actinin in Caco-2 cells. However, integrin β1 antibody blocking or KO reduced the levels of these proteins in infected cells (Figures 3A–D). Immunofluorescence staining further showed that vinculin content was increased during *E. coli* O157:H7 infection, while integrin β1 KO impaired the accumulation of vinculin in response to infection (Figure 3E). These data collectively showed that integrin β1 was an important factor that mediated host FAs recruitment and assembly in response to *E. coli* O157:H7 infection.

**FIGURE 4:**
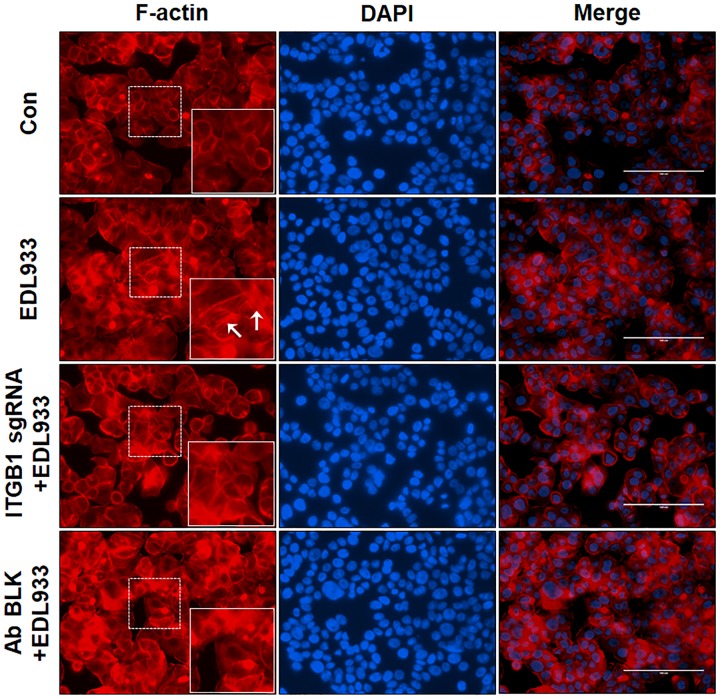
Integrin β1 was associated with *E. coli* O157:H7-mediated actin polymerization. Immunofluorescence staining for F-actin in Caco-2 cells. Caco-2 cells were immuno-blocked with integrin β1 antibody (Ab BLK) or transfected with integrin β1 sgRNA (ITGB1 sgRNA) plasmid followed by infection with EDL933 strain for 4 h. Cells were then subjected to phalloidin-TRIC staining. Con, Caco-2 cells without infection; EDL933, Caco-2 cells infected with EDL933 strain. ITGB1 sgRNA+EDL933, integrin β1 knockout Caco-2 cells infected with EDL933 strain; Ab BLK+EDL933, integrin β1 blocked Caco-2 cells infected with EDL933 strain. Blue, DAPI; Red, F-actin; Images were taken at 200 × magnification.

The assembly of integrins and FAs serve as a platform for the organization of actin filaments. *E. coli* O157:H7 attachment to host cells is typically associated with actin rearrangement. When integrin β1 was KO or blocked by antibody, the actin polymerization induced by infection was subsided (Figure 4), showing that integrin β1 was also implicated in infection-induced actin polymerization.

**FIGURE 5:**
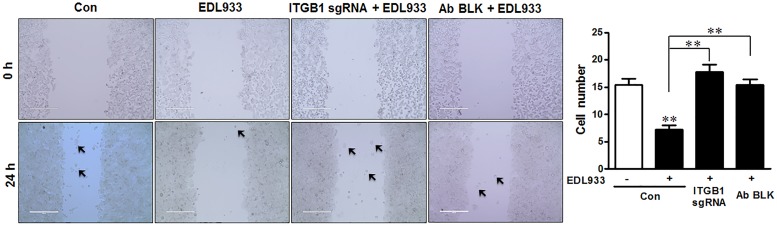
*Escherichia coli* O157:H7 inhibited cell migration. Caco-2 cell monolayers were scratched and followed by infection with EDL933 strain for 4 h. The migrated cells were counted 24 h post infection at 37°C. Con, cells without infection; EDL933, cells infected with EDL933 strain. ITGB1 sgRNA+EDL933, integrin β1 knockout cells infected with EDL933 strain; Ab BLK+EDL933, integrin β1-blocked cells infected with EDL933 strain. Images were taken at 100 × magnification. Means ± SEM; *n* = 4. ^∗∗^*P* ≤ 0.01.

Enhanced FA assembly and decreased FAK activation could lower the ability of cell migration (Sieg et al., 1999; Kim et al., 2009). Consistent with enhanced FA assembly, *E. coli* O157:H7 infection significantly inhibited cell migration during wound healing. This inhibition phenomenon was attenuated by integrin β1 KO or antibody blocking (Figure 5).

### Intimin Is Involved in FAK Inhibition and FA Accumulation

**FIGURE 6:**
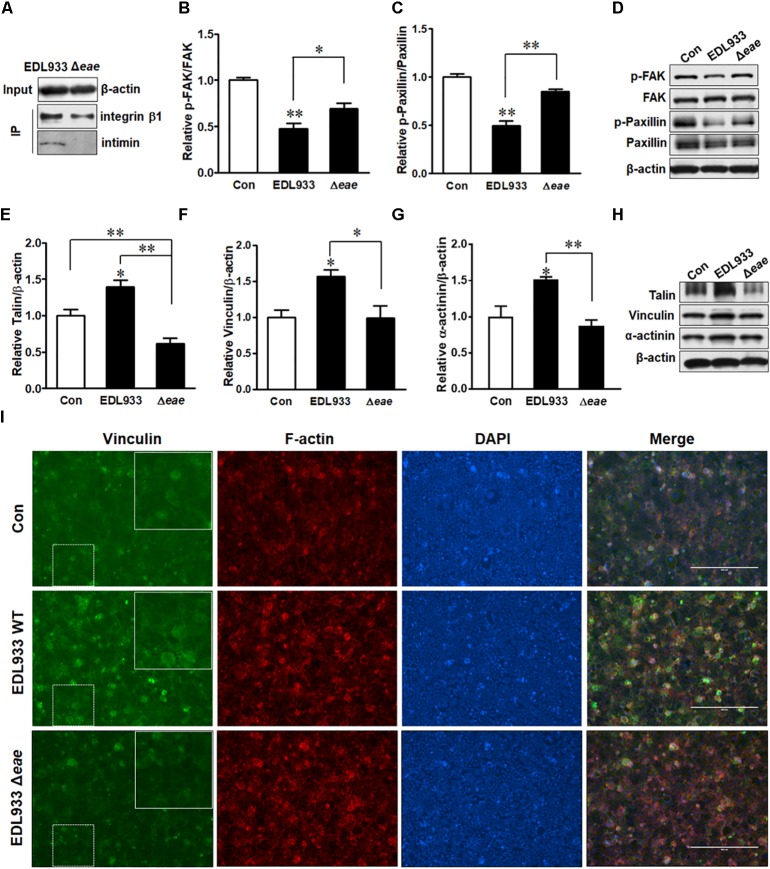
Intimin was associated with focal adhesion alterations. **(A)** Co-immunoprecipitation of integrin β1 and intimin interaction. Input, the whole cell lysate detected with β-actin antibody; IP, the immunoprecipitated lysates were immunoblotted with integrin β1 or intimin antibodies. **(B–D)** Phosphorylation of FAK and paxillin. **(E–H)** Immunoblotting analysis of talin, vinculin, and α-actinin. **(I)** Immunofluorescence staining for vinculin in Caco-2 cells at 4 h post-infection with EDL933 strain or EDL933 intimin mutant strain (Δ*eae*). Con, cells without infection; EDL933, cells infected with EDL933 strain; Δ*eae*, cells infected with EDL933 intimin mutant strain. Blue, DAPI; Green, vinculin. Images were taken at 100 × magnification. Means ± SEM; *n* = 4. ^∗∗^*P* ≤ 0.01; ^∗^*P* ≤ 0.05.

Immunoprecipitation assay indicated that intimin was associated with integrin β1 (Figure 6A). To further understand the role of intimin in host FA formation, we infected Caco-2 cells with intimin mutant strain (Δ*eae*). Infection with Δ*eae* strain did not suppress FAK and paxillin (Figures 6B–D), indicating a regulatory role of intimin in host signaling transduction. Consistently, FA proteins including talin, vinculin and α-actinin were not altered in cells infected with Δ*eae* strain (Figures 6E–H). Immunofluorescent staining further showed that Δ*eae* resulted in a lesser accumulation of vinculin as compared with EDL933 WT infected cells (Figure 6I). Interestingly, our data also showed that the *tir* deletion mutant (Δ*tir*) strain was incapable of causing dephosphorylation of FAK and paxillin (Supplementary Figure S3). The cytoplasmic C and N-terminus of Tir bind to FA proteins such as talin, vinculin, and α-actinin (Freeman et al., 2000; Huang et al., 2002), which might interfere with FAK activity. The interaction between Tir and host FA may strengthen its association with host cell surface and facilitate colonization.

### Quercetin Inhibited *E. coli* O157:H7 Adherence Associated With Decreased Integrin β1 Expression and FA Formation

**FIGURE 7:**
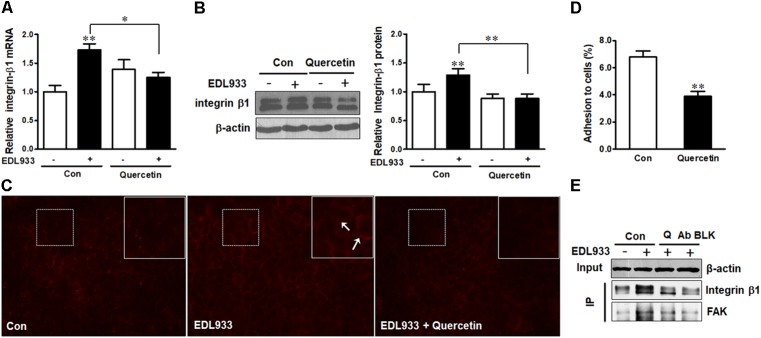
Quercetin reduced *E. coli* O157:H7 adhesion and prevented integrin β1 overexpression induced by *E. coli* O157:H7 infection. **(A,B)** mRNA expression and protein level of integrin β1. **(C)** Immunofluorescence staining of integrin β1. Red, integrin β1. Images were taken at 200 × magnification. **(D)** Adhesion of EDL933 to Caco-2 cells treated with or without quercetin. **(E)** Co-immunoprecipitation analysis of integrin β1 and FAK interaction. Input, the whole cell lysate detected with β-actin; IP, the immunoprecipitated lysates were detected with integrin β1 or FAK antibody. Caco-2 cells were pretreated with 0 or 200 μM quercetin for 12 h, then followed by infection with EDL933 strain for 4 h when cells were collected for respective assays. Means ± SEM; *n* = 4. ^∗∗^*P* ≤ 0.01; ^∗^*P* ≤ 0.05.

**FIGURE 8:**
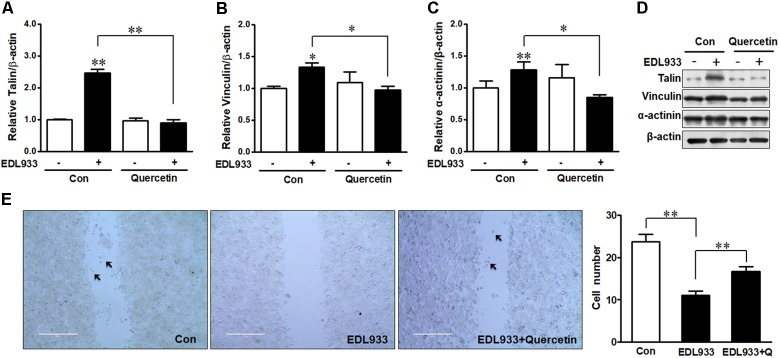
Focal adhesions were reduced by quercetin upon *E. coli* O157:H7 infection. **(A–D)** Immunoblotting analysis of talin, vinculin, and α-actinin. **(E)** Migration of Caco-2 cells pretreated with or without quercetin followed by infection with EDL933 strain for 4 h. Con, cells without infection; EDL933, cells infected with EDL933 strain. EDL933+Quercetin: cells pretreated with quercetin then infected with EDL933 strain. Means ± SEM; *n* = 4. ^∗∗^*P* ≤ 0.01; ^∗^*P* ≤ 0.05.

Quercetin reduced *E. coli* O157:H7-induced inflammasome activation (Xue et al., 2017). Here, we further showed that quercetin prevented integrin β1 expression (Figure 7A) and protein content (Figures 7B,C) in Caco-2 cells infected with EDL933, associated with decreased adhesion to Caco-2 cells (Figure 7D). Furthermore, quercetin attenuated the association of FAK with integrin β1 in EDL933 infected cells (Figure 7E). Additionally, quercetin reduced the protein contents of talin, vinculin, and α-actinin that were increased due to *E. coli* O157:H7 infection (Figures 8A–D), and rescued cell migration inhibited by *E. coli* O157:H7 (Figure 8E).

## Discussion

### Integrin β1 Is a Potential Receptor for *E. coli* O157:H7 Adhesion

Integrins are a large family of heterodimeric receptors that are associated with a wide range of cell-to-cell interactions (Hynes, 1992). Integrin α5β1 is the most expressed and best characterized integrin heterodimer and functions as a receptor for many bacteria, such as *Shigella flexneri* and *Pseudomonas aeruginosa* (Watarai et al., 1996; Roger et al., 1999). The adhesin protein, CagL of *Helicobacter pylori* binds to and activates integrin α5β1 receptor and induces intracellular signaling (Kwok et al., 2007). Notably, many pathogenic bacteria enhance the surface level of integrins. *H. pylori*-infected gastric epithelial cells have a higher expression of both integrin α5 and β1 (Cho et al., 2006), and *S. flexneri* infection increases integrin β1 in HeLa cells (Kim et al., 2009). Consistently, our data also showed that both integrin α5 and β1 were upregulated in *E. coli* O157:H7-infected cells as compared to non-infected cells. Integrin β1 KO or blocking by integrin β1 antibody decreased bacterial attachment, indicating that integrin β1 was involved in *E. coli* O157:H7 adhesion.

### Inhibition of FAK May Strengthen Bacterial Colonization

Accumulating evidence shows that virulence factors of pathogens can utilize host kinases to manipulate host signaling. OspE, an effector of type III secretion system (T3SS) of *Shigella* (Miura et al., 2006), interacts ILK and subsequently reduces the phosphorylation of FAK and paxillin (Kim et al., 2009), resulting in stabilization of FAs and attenuated cell turnover (Miura et al., 2006). EspO1-1, a homolog of OspE in *E. coli* O157:H7 (Kim et al., 2009; Morita-Ishihara et al., 2013), similarly interacts with FAK to stabilize FA complex and inhibit the detachment of host cells from the ECM (Morita-Ishihara et al., 2013), indicating *E. coli* O157:H7 also has the ability to counteract the exfoliation of epithelial cells, which benefits its persistence. In our study, we found that intimin was co-immunoprecipitated with integrin β1, while intimin mutant strain was unable to induce FAK and paxillin dephosphorylation, suggesting that intimin mediates FAK and FA activity, and has ability to interact with integrin β1 to exploit host outside-in signaling.

**FIGURE 9:**
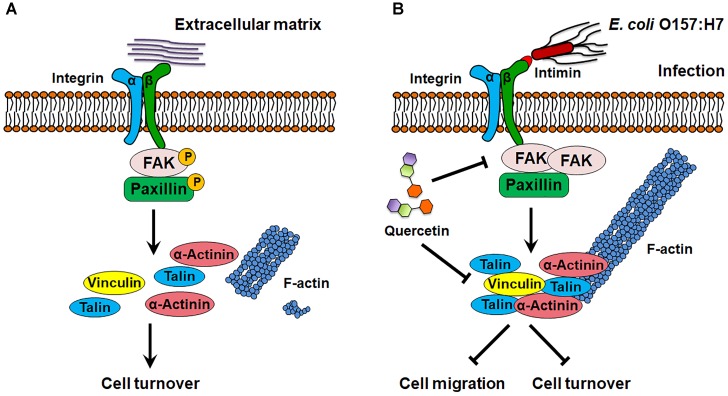
Schematic diagram of *E. coli* O157:H7 colonization on host cell. **(A)** In normal state, FAK is activated by integrin β1, which promotes focal adhesion turnover and cell motility. **(B)** *E. coli* O157:H7 can interact with host integrin β1, which recruits FAK and paxillin, and inhibits their activation, thereby stabilizes focal adhesion and increases actin polymerization. Quercetin prevents integrin β1 overexpression and focal adhesion formation as well as the association between integrin β1 and FAK induced by infection. FAK, focal adhesion kinase. → Activate or promote, 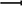 Inhibit.

Integrins transduce extracellular signals into the host cells through association with intracellular adaptor proteins and protein kinases such as FAK (Dia and Gonzalez de Mejia, 2011) and ILK (Gagne et al., 2010). These kinases serve as docking sites for recruitment of other kinases and FA components such as paxillin, talin, vinculin, and mediate cytoskeletal reorganization (Schaller et al., 1995). FAK activation induces disassembly of FAs and correlates with enhanced cell turnover (Webb et al., 2004; Hamadi et al., 2010). In FAK^-/-^ cells, the disassembly of FAs is significantly impaired with attenuated cell mobility (Webb et al., 2004). We found that infection enhanced interaction between FAK and integrin β1, which inhibits the phosphorylation of FAK and subsequently deactivates paxillin, thereby causing FA accumulation (Figure 9). As a result, cell migration was reduced in response to *E. coli* O157:H7 infection, which may inhibit host shedding and turnover. In support of our finding, FAK activation promotes migration of both endothelial cells and fibroblasts (Zhao and Guan, 2011), while FAK deficiency decreases cell migratory activity (Zhao and Guan, 2011) with an increased formation of FAs (Ilic et al., 1995).

### Quercetin Decreases Bacterial Infection by Regulating Integrin β1

Quercetin decreases ECM components such as collagen III productions and assembly in human corneal fibroblasts (McKay et al., 2015), and decreases cell surface level of integrin β1 in different cell types (He et al., 2015; Doersch and Newell-Rogers, 2017). In this study, although quercetin did not alter integrin β1 expression in uninfected cells, quercetin prevented the increase of both integrin β1 and integrin α5 expressions, as well as FA protein assembly induced by infection. Mechanisms for such preventive effects are twofold. Quercetin could directly interfere with integrin signaling elicited by bacteria to suppress FA accumulation and bacterial attachment, or the reduced bacterial attachment due to quercetin proportionally weakened intracellular signaling in comparison to untreated cells with more bacterial attachment. These data collectively suggested that quercetin prevented *E. coli* O157:H7 adhesion to epithelial cells through attenuation of integrin β1 accessibility to bacteria and/or suppression of intracellular signaling. The resultant effect may contribute to the reduced FA assembly.

In summary, *E. coli* O157:H7 attached to epithelial cells partially through the interaction with host integrin β1, which inhibited FAK phosphorylation and stabilized FA formation.

Quercetin inhibits bacterial infection likely via attenuated association between integrin β1 and FAK. Given that antibiotics are not applicable for *E. coli* O157:H7 infection, these data provide a potential therapeutic application of quercetin for minimizing and eliminating *E. coli* O157:H7 infection. These data also suggest a broad application of polyphenolic compounds in the prevention of enteric pathogenic infection. However, additional *in vivo* studies to test the effects of quercetin on inhibiting *E. coli* O157:H7 infection will further strengthen our conclusions.

## Author Contributions

YX, MD, and M-JZ designed the study, analyzed the data, and reviewed the manuscript. YX conducted the experiments. YX drafted the manuscript. MD and M-JZ revised the manuscript.

## Conflict of Interest Statement

The authors declare that the research was conducted in the absence of any commercial or financial relationships that could be construed as a potential conflict of interest.
