# Supplementary material for: Quercetin Prevents Escherichia coli O157:H7 Adhesion to Epithelial Cells via Suppressing Focal Adhesions
Source: Front Microbiol. 2019 Jan 16;9:3278. doi: 10.3389/fmicb.2018.03278 (PMC6343519; doi:10.3389/fmicb.2018.03278)
Supplement: Supplementary file 1 [file Table_1.docx]

**SUPPLEMENTARY INFORMATION**

**Method and material**

***Bacterial survival assay.*** *E. coli* O157:H7 was grown in LB broth at 37 °C overnight with aeration. The overnight bacterial culture was subcultured in LB broth with 0 or 200 μM quercetin for up to 12h at 37°C, sampled at 0, 4, 8, 12 h post-incubation. Bacterial samples were serially diluted and appropriate dilutions were plated on LB agar plates. The colonies were counted after 24 h incubation at 37 ºC.

**Table S1. Primer sets used for quantitative RT-PCR in Caco-2 cells**

| **Gene Name** | **Accession No.** | **Product Size** | **Direction** | **Sequence (5’🡪3’)** | **Source** |
| --- | --- | --- | --- | --- | --- |
| *Itgb1* | NM_002211.3 | 252bp | Forward | CCGCGCGGAAAAGATGAAT | This study |
|  |  |  | Reverse | ATGTCATCTGGAGGGCAACC |  |
| *Itga5* | NM_002205.3 | 123bp | Forward | CAAGACTTTCTTGCAGCGGG | This study |
|  |  |  | Reverse | GCCACCTGACGCTCTTTTTG |  |
| ***Actb*** | NM_001101.3 | 100bp | Forward | GATGAGATTGGCATGGCTTT | [1] |
|  |  |  | Reverse | CACCTTCACCGTTCCAGTTT |  |

**
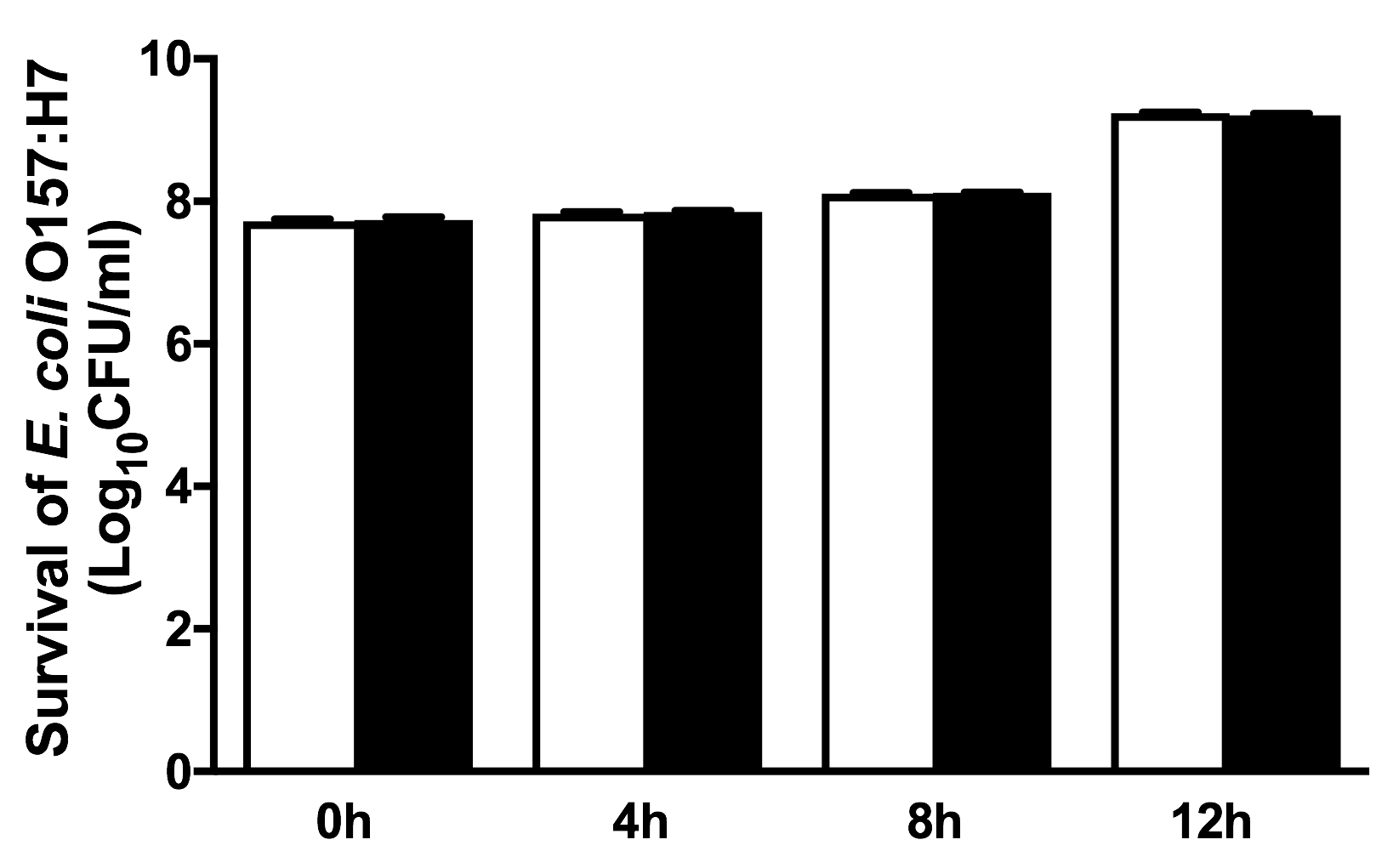
Supplementary Figure 1.**

**Figure S1. Quercetin did not affect *E. coli* O157:H7 survival or growth.** *E. coli* O157:H7 was incubated in LB broth with 0 or 200 μM quercetin at 37°C for indicated time. Means ± SEM; n = 4.

**Supplementary Figure 2.**

**
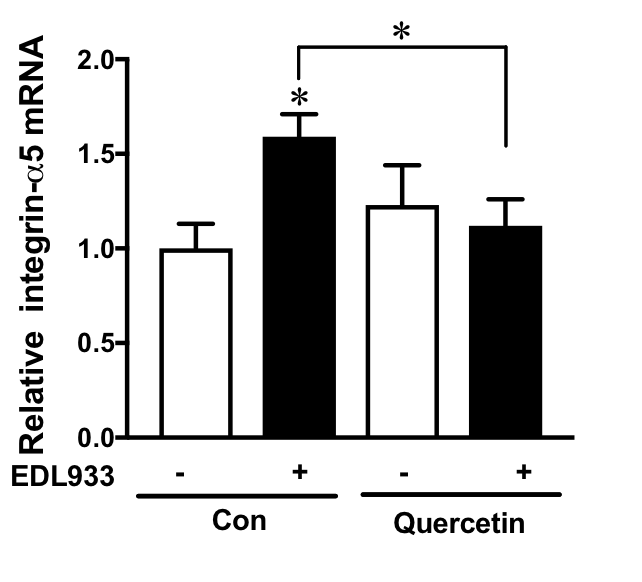
**

**Figure S2. Quercetin prevented *E. coli* O157:H7-induced mRNA expression of integrin α5.** Caco-2 cells were pretreated with 0 or 200 μM quercetin for 12 h, followed by infection with *E. coli* O157:H7 EDL933 strain for 4 h. Means ± SEM; n = 4. *, *P* < 0.05.

**Supplementary Figure 3.**

**Figure S3. Tir was involved in FAK manipulation. A-C** Phosphorylation of FAK and paxillin in control Caco-2 cells (Con) or cells infected with or without *E. coli* O157:H7 EDL933 WT strain (EDL933), Tir knockout strain (Δ*tir*) or *tir* complementation strain (Δ*tir*+p*tir*). Means ± SEM; n = 4. **, *P* < 0.01; *, *P* < 0.05.

**Reference:**

1. **Xue Y, Zhang H, Sun X*, et al.*** Metformin improves ileal epithelial barrier function in interleukin-10 deficient mice. *Plos One*. 2016; 11: e0168670.
